# Setting and Monitoring of Mechanical Ventilation During Venovenous ECMO

**DOI:** 10.1186/s13054-023-04372-2

**Published:** 2023-03-21

**Authors:** Benjamin Assouline, Alain Combes, Matthieu Schmidt

**Affiliations:** 1 Médecine Intensive Réanimation, Institut de Cardiologie, Assistance Publique-Hôpitaux de Paris, Paris, France; 2 Sorbonne Université, GRC 30, RESPIRE, UMRS 1166, ICAN Institute of Cardiometabolism and Nutrition, Paris, France

## Abstract

This article is one of ten reviews selected from the Annual Update in Intensive Care and Emergency Medicine 2023. Other selected articles can be found online at https://www.biomedcentral.com/collections/annualupdate2023. Further information about the Annual Update in Intensive Care and Emergency Medicine is available from https://link.springer.com/bookseries/8901.

## Introduction

In patients with the acute respiratory distress syndrome (ARDS), mechanical ventilation can cause ventilator-induced lung injury (VILI) through multiple mechanisms, including volutrauma, barotrauma, atelectrauma, myotrauma, and biotrauma [1]. In the most severe forms of ARDS, the smaller the baby lung, the greater the potential for unsafe ventilation despite mechanical ventilation volume and pressure limitation. To further limit the energy transmitted to the lungs by the mechanical ventilator, “ultra-lung-protective” ventilation reducing tidal volume (≤ 4 ml/kg), respiratory rate (< 20/min), and airway (plateau pressure < 25 cmH_2_O and driving pressure ≤ 15 cmH_2_O) pressures has been proposed [2]. However, this strategy can result in severe respiratory acidosis without extracorporeal gas exchange using extracorporeal life support (ECLS) devices. Venovenous extracorporeal membrane oxygenation (VV-ECMO) is a form of ECLS that provides full extracorporeal blood oxygenation and carbon dioxide removal, which can replace pulmonary function. VV-ECMO allows marked reductions in tidal volume, respiratory rate, plateau and driving pressures [3, 4]. It has been associated with survival benefits in randomized controlled trials (RCTs) and meta-analyses [3–6]. However, optimal mechanical ventilation settings on ECMO are still debated. In this narrative review, we summarize the current knowledge, rationale, and evidence for mechanical ventilation management and monitoring in patients receiving VV-ECMO for severe ARDS. We will also discuss the research agenda in this field.

## Historical Perspective

### Ventilation Strategies in ECMO Landmark Trials

There is a paucity of data regarding optimal mechanical ventilation settings during ECLS (Table 1). Current recommendations are thus based on expert opinion [7] and the results of very few landmark trials [3, 4]. The concept of lung rest during ECLS was first proposed by Gattinoni et al. in a non-controlled series [8], in which ARDS patients were ventilated with peak inspiratory pressures limited to less than 35–45 cmH_2_O, low respiratory rates (< 5/min), and positive end-expiratory pressure (PEEP) set to 15–25 cmH_2_O. In the CESAR trial [4], patients were randomized to receive either conventional management at their center (90 patients) or to be referred for consideration for ECMO at an ECMO center (90 patients), where a “lung-rest” strategy was applied under ECMO (pressure control mode, peak inspiratory pressure limited to 20–25 cmH_2_O, PEEP 10–15 cmH_2_O, respiratory rate 10/min, and fraction of inspired oxygen [FiO_2_] 0.3). Although the rate of mortality or severe disability was lower at 6 months in the ECMO group, the study was criticized for several methodological limitations. Specifically, only 75% of the referred patients received ECMO, and protective mechanical ventilation was applied in only 70% of the control group. In the EOLIA trial [3], patients with severe ARDS were randomly assigned to receive immediate VV-ECMO or conventional protective mechanical ventilation. Ultra-protective ventilation was provided to the ECMO group either by assisted-control mode (tidal volume reduced to obtain plateau pressure ≤ 24 cmH_2_O, PEEP ≥ 10 cmH_2_O, respiratory rate 10 to 30 cycles/min, and FiO_2_ 0.3) or by airway pressure release ventilation (APRV; high pressure ≤ 24 cmH_2_O, PEEP ≥ 10 cmH_2_O, ratio of inspiratory to expiratory time 1:2, and FiO_2_ 0.3). In the hours following randomization, ECMO patients had a significant decrease in tidal volume (6.0 ± 1.3 vs. 3.5 ± 1.0 ml/kg), plateau pressure (30 ± 6 vs. 24 ± 3 cmH_2_O), driving pressure (18 ± 7 vs. 13 ± 2 cmH_2_O), respiratory rate (30 ± 5 vs. 23 ± 2 breaths/min), while PEEP (12 ± 4 vs. 11 ± 3 cmH_2_O) remained unchanged. Mortality was lower in the ECMO group (35% vs. 46%) although this difference did not reach statistical significance (p = 0.07).

**Table 1:** Mechanical ventilation settings in landmark trials and cohorts in patients with acute respiratory distress syndrome (ARDS) treated with venovenous-extracorporeal membrane oxygenation (VV-ECMO)

	Design	Number of patients	Mechanical ventilation strategy on ECMO	Mean mechanical power reduction	Main findings
CESAR [4]	RCT	180	**PC mode with:** • PIP 20–25 cmH_2_O • PEEP 10–15 cmH_2_O • RR 10/min • FiO_2_ 0.3	Not available	Referral to an ECMO center for severe ARF (Murray score > 3.0 or pH < 7.20): • Improves survival without severe disability (RR 0.69; 95% CI 0.05–0.97, p = 0.03) • Cost-effective strategy
EOLIA [3]	RCT	249	**ACV mode with:** • V_T_ to obtain Pplat ≤ 24 cmH_2_O • PEEP ≥ 10 cmH_2_O • RR 10 to 30/min • FiO_2_ 0.3 **APRV mode with:** • High pressure ≤ 24 cmH_2_O • Low pressure ≥ 10 cmH_2_O FiO_2_ 0.3	From 28 to 10 J/min	On day 60: • 11% absolute mortality reduction in favor of the ECMO group (35% vs. 46%, p = 0.07) • 28% of the control group required crossover and emergent cannulation
LIFEGARDS [10]	Prospective cohort	350	• V_T_ 3.7 ± 2.0 ml/kg • Pplat 24 ± 7 cmH_2_O • ΔP 14 ± 4 cmH_2_O • RR 14 ± 6/min	From 26 to 6.6 J/min	A combination of V_T_ (≤ 4 ml/kg) and a ΔP ≤ 15 cmH_2_O during the first two days of ECMO was obtained in 45% of patients Lack of association between mechanical ventilation settings during the first two days of ECMO and survival
Serpa Neto et al. [19]	Meta-analysis	545	• V_T_ 4.0 ± 1.7 ml/kg PBW • Pplat 26.2 ± 4.6 cmH_2_O • ΔP 13.7 ± 5.3 cmH_2_O • RR 17.8 ± 8/min	Not available	In hospital mortality = 35.2% ΔP was the only ventilatory parameter that showed an independent association with in-hospital mortality
Wang et al.[32]	RCT	104	• V_T_ 4.0 ± 1.3 ml/kg PBW • Pplat 24.0 ± 2.6 cmH_2_O • PEEP 13.1 ± 2.4 cmH_2_O • RR 17.7 ± 4.8/min	From 26 to 7.5 J/min	The Ptp-guided group had: • Higher rate of successful weaning (p = 0.017) • Lower 60-day mortality rate compared to the lung rest group (32.7% vs. 54%, p = 0.030) • Shorter ECMO duration (p = 0.004)

*RCT* randomized controlled trial, *PC* pressure-control, *ACV* assist-control ventilation, *APRV* airway pressure release ventilation, *PIP* peak inspiratory pressure, *V*_*T*_ tidal volume, *Pplat* plateau pressure, *PBW* predicted body weight, *PEEP* positive end-expiratory pressure, *Ptp* transpulmonary pressure, *RR* respiratory rate, *ΔP* driving pressure, *FiO*_*2*_ the fraction of inspired oxygen, *ARF* acute respiratory failure

### Current Practice in ECMO-Experienced Centers

An international cross-sectional survey [9] conducted in 2013 among 141 medical directors and ECMO program coordinators from 283 Extracorporeal Life Support Organization (ELSO)-registered centers, revealed that only 27% of centers had an explicit mechanical ventilation protocol for patients on VV-ECMO. The majority of these centers (77%) reported “lung rest” to be the primary goal of mechanical ventilation, whereas 9% reported “lung recruitment” to be their ventilation strategy. A tidal volume of 6 ml/kg or less was targeted by 76% of respondents, but only 34% of them were setting tidal volumes to less than 4 ml/kg. PEEP was ≤ 10 cmH_2_O in 77% of the patients. More recently, the LIFEGARDS (ventiLatIon management oF patients with Extracorporeal membrane oxyGenation for Acute Respiratory Distress Syndrome) was the first prospective study specifically designed to describe the ventilatory management of ECMO-treated patients with ARDS [10]. LIFEGARDS included an international, multicenter cohort of 350 patients supported by ECMO in 23 medium- to high-volume ECMO intensive care units (ICUs) across 10 countries. It confirmed the widespread adoption of ultra-protective ventilation after ECMO initiation, with marked reduction in tidal volume (6.4 ± 2.0 vs. 3.7 ± 2.0 ml/kg), plateau pressure (32 ± 7 vs. 24 ± 7 cmH_2_O), driving pressure (20 ± 7 vs. 14 ± 4 cmH_2_O), respiratory rate (26 ± 8 vs. 14 ± 6 breaths/min), and mechanical power (26.1 ± 12.7 vs. 6.6 ± 4.8 J/min), while PEEP (12 ± 4 vs. 11 ± 3 cmH_2_O) was kept greater than 10 cmH_2_O in most patients. No association was however found in multivariable analysis between ventilator settings during the first 2 days of ECMO and survival.

## Targeting Ultra-Lung-Protective Mechanical Ventilation During ECMO

### Tidal Volume

Decreasing tidal volume is the cornerstone of limiting the stress and strain applied by the mechanical ventilator to the lungs and the resulting VILI. Using a rat model of acid-induced lung injury, a tidal volume reduction from 12 to 6 to 3 ml/kg, with the same level of PEEP (10 cmH_2_O), decreased pulmonary edema and lung injury and increased protection of the alveolar epithelium [11]. Indeed, the limited tidal volume reduction (6.3 to 4.5 ml/kg) due to insufficient CO_2_ removal to control respiratory acidosis by the extracorporeal carbon dioxide removal (ECCO_2_R) device may explain the failure of the REST trial to improve the outcomes of ARDS patients [12]. By contrast, ECMO enabled larger tidal volume reduction (< 4 ml/kg) in patients randomized to the ECMO group of the EOLIA trial and in those of the LIFEGARDS cohort. Targeting a tidal volume of less than 4 ml/kg is recommended in the guidelines of the Extracorporeal Life Support Organization (ELSO) [7].

### Plateau Pressure

Plateau pressure is easily measurable at the bedside and received considerable attention after publication of the ARMA trial [13]. The REVA Network study on H_1_N_1_ influenza-related ARDS reported that the mean plateau pressure after initiation of VV-ECMO was significantly lower in survivors than in non-survivors (25 ± 3 vs. 29 ± 5 cmH_2_O; p < 0.01) [14]. In that study, higher plateau pressures (>25 cmH_2_O) on the first day of VV-ECMO were significantly associated with mortality (odds ratio [OR] = 1.33, 95% confidence interval [CI] 1.14 to 1.59, p < 0.01). A plateau pressure <25 cmH_2_O was targeted in the most recent VV-ECMO series [15, 16] and is also recommended by the ELSO [7].

### Driving Pressure

The driving pressure is the plateau airway pressure minus PEEP. It can also be expressed as the ratio of tidal volume to respiratory system compliance (ΔP = V_T_/C_RS_), indicating the decreased functional size of the lung observed in patients with ARDS (i.e., baby lung). Driving pressure is a strong predictor of mortality in patients with ARDS as demonstrated by a *post hoc* analysis of previous RCTs and subsequent studies [10, 17, 18], with driving pressure >14 cmH_2_O being associated with a higher risk of mortality [17]. An individual patient data meta-analysis of observational studies in adult patients with ARDS receiving ECMO reported that driving pressure was the only ventilatory parameter that showed an independent association with in-hospital mortality [19]. In that context, targeting a driving pressure <14 cmH_2_O on VV-ECMO appears desirable and is currently applied in centers with high ECMO volume [10].

### Respiratory Rate

The frequency of lung collapse and expansion, i.e., the respiratory rate, contributes to VILI. In a pig model of ARDS, Grasso et al. assessed the benefit of respiratory rate reduction combined with ECCO_2_R [20]. At a fixed tidal volume (6 ml/kg), lower respiratory rate was associated with reduced biotrauma while lung aeration was preserved [20]. A secondary analysis of the LUNG SAFE study [21] also confirmed that higher respiratory rate was independently associated with increased in-hospital mortality. More recently, Costa et al. demonstrated, in a retrospective pooled database of 4549 patients with ARDS, that only the driving pressure and respiratory rate had significant associations with mortality [22]. In that study, the impact of the driving pressure on mortality was four times as large as that of the respiratory rate. While the ELSO recommends a respiratory rate of 4–15 breaths/min [7], higher respiratory rates on ECMO were reported in EOLIA (23 ± 2) [3] and in the LIFEGARDS study (14 ± 6) [10]. A minimal respiratory rate (4/min) may however be needed to maintain lung volume and to avoid derecuitement during ultra-lung-protective ventilation [8].

### Mechanical Power

**Fig. 1:**
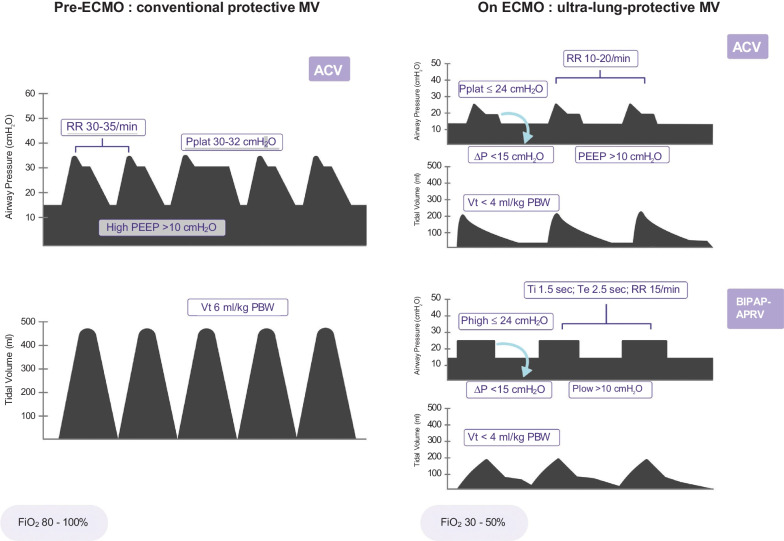
Pre-extracorporeal membrane oxygenation (ECMO) conventional protective ventilation compared to ultra-lung protective mechanical ventilation during ECMO. *ACV* assist-control ventilation, *BIPAP-APRV* bilevel positive airway pressure-airway pressure release ventilation, *RR* respiratory rate, *PEEP* positive end-expiratory pressure, *PBW* predicted body weight, *Vt* tidal volume, *FiO*_*2*_ inspired fraction in O_2_, *Pplat* plateau pressure, *∆P* driving pressure, *P*_*high*_ high pressure, *P*_*low*_ low pressure, *Ti* inspiratory time, *Te* expiratory time

Mechanical power represents the energy delivered by the ventilator to the respiratory system [23]. It is a function of transpulmonary pressure, tidal volume, and respiratory rate and was shown to be independently associated with mortality in ARDS patients when > 17 J/min [24]. By applying ultra-lung-protective ventilation during ECMO, the mechanical power can be dramatically reduced. Indeed, it was significantly lower (10 J/min vs. 28 J/min) in the ECMO compared to the control group in the EOLIA trial, an effect mediated by a 43% and 23% reduction in tidal volume and respiratory rate, respectively [25]. Similarly, the mean mechanical power was reduced from 26 J/min to 6.6 J/min after ECMO initiation in the LIFEGARDS cohort study [10]. Although the mechanical power concept has several limitations, it may enable the contribution of all modifiable mechanical ventilation settings (tidal volume, respiratory rate, driving pressure, PEEP, inspiratory to expiratory ratio, inspiratory flow) to VILI to be quantified. Although its computation may help to guide current practice (Fig. 1), the extent to which mechanical power should be reduced in ECMO patients remains undetermined.

### Applying Apneic Ventilation?

Decreasing tidal volume to less than 4 ml/kg may not be sufficient to prevent excess strain (defined as tidal volume/end-expiratory lung volume) delivered by mechanical ventilation to inflamed and inhomogeneous lungs, as recently suggested in a randomized crossover physiological study in 10 patients with ARDS receiving VV-ECMO [18]. In this study, a substantial risk of biotrauma and VILI persisted despite a mean tidal volume of 2.4 ml/kg in patients with low respiratory system compliance. Specifically, a linear relationship existed between changes in inspiratory pressure and concentrations of plasma biomarkers (soluble receptor for advanced glycation endproducts [S-RAGE], interleukin [IL]-6, tumor necrosis factor [TNF]-alpha) during mechanical ventilation. Biotrauma was lowest in the absence of tidal ventilation in the continuous positive airway pressure (CPAP) mode (10 cmH_2_O). Similarly, Graf et al. compared lung protective with apneic ventilation in 24 patients with severe ARDS receiving VV-ECMO in a prospective, monocenter physiological study [26]. Ultra-lung-protective ventilation was associated with increased stress, strain, and mechanical power, despite a low driving pressure (11.9 ± 5.8 cmH_2_O). In a large animal model of ARDS supported with VV-ECMO, near apneic ventilation (driving pressure 10 cm H_2_O, PEEP 10 cm H_2_O, and respiratory rate 5/min) was also associated with decreased lung injury and fibroproliferation compared to a conventional ventilation strategy [27]. Although (near) apneic ventilation might be the ultimate strategy to decrease VILI during ECMO, more data and larger studies on patient-centered outcomes are now needed before it can be widely adopted. Limitations of near apneic ventilation should also be evaluated. The absence of lung cycling may have short- and long-term physiological consequences and may require deeper sedation and sometimes continuous neuromuscular blockade to control the respiratory drive and subsequent patient self-inflicted lung injury (P-SILI). The technique also requires higher blood flow in the VV-ECMO circuit to reach adequate oxygenation, which may be associated with complications such as hemolysis.

### Preserving Spontaneous Ventilation and Diaphragmatic Function to Minimize P-SILI?

Preserving diaphragmatic function by allowing spontaneous respiratory movements may facilitate weaning from mechanical ventilation, as short periods (18 to 69 h) of diaphragm inactivity on mechanical ventilation were associated with a 55% decrease in transdiaphragmatic pressure and marked atrophy of both slow-twitch and fast-twitch diaphragm fibers in humans [28]. On the other hand, spontaneous breathing could be associated with strong respiratory efforts and elevated transpulmonary pressure in patients with high respiratory drive and low pulmonary compliance and cause P-SILI [29, 30]. Although switching from controlled to assisted-spontaneous ventilation has several benefits (muscle function preservation, decreased sedation, hemodynamic improvement), minimizing P-SILI while maintaining (part of) the diaphragm activity is challenging in patients with the most severe forms of ARDS receiving ECMO. In that context, the APRV mode that combines the control of plateau and driving pressures while allowing non-synchronized spontaneous breathing may be valuable.

## How to Set the Optimal PEEP on ECMO?

**Fig. 2:**
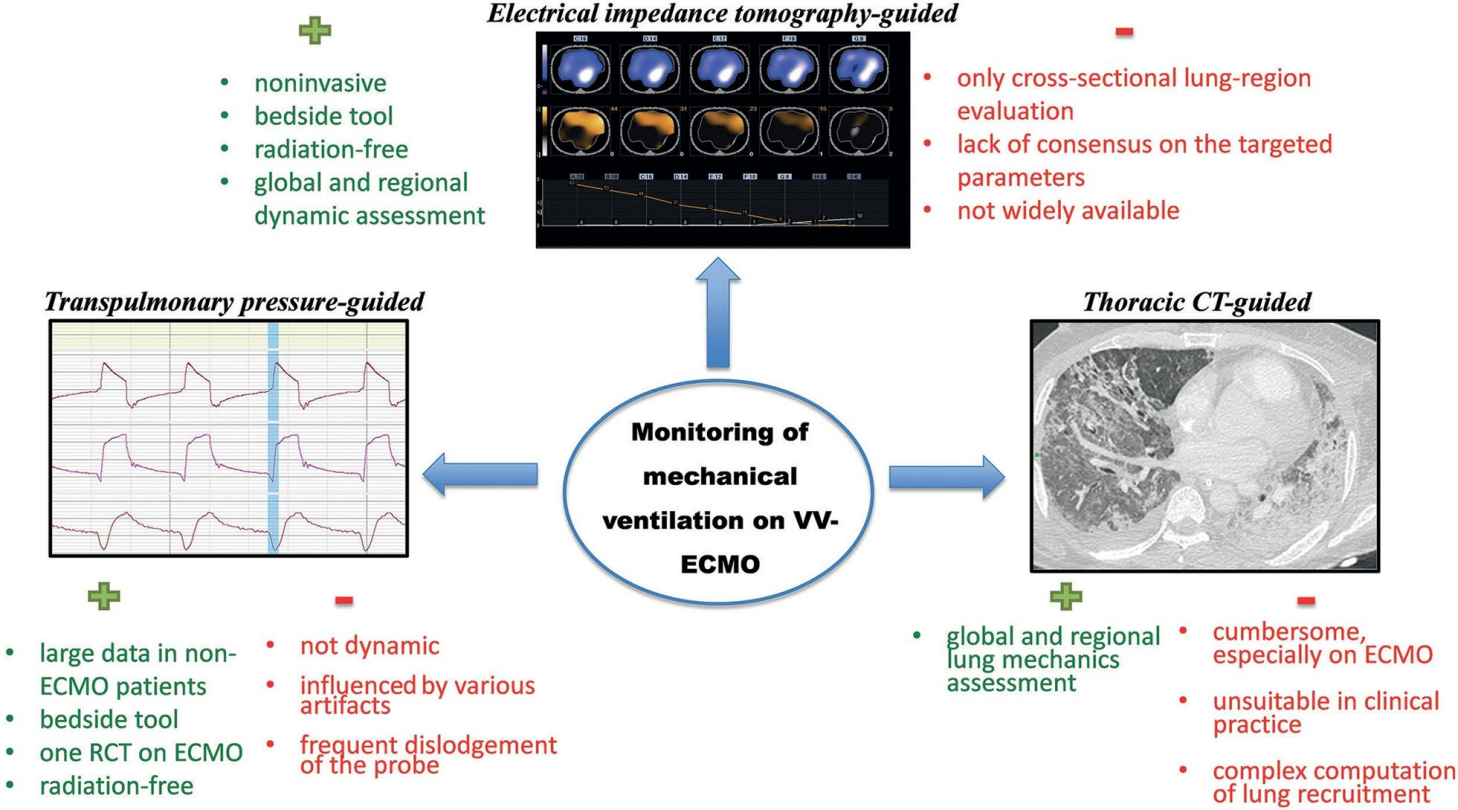
Tools to set positive end-expiratory pressure on venovenous extracorporeal membrane oxygenation (VV-ECMO). *RCT* randomized controlled trial, *CT* computed tomography

As with any intervention, the ultra-lung-protective ventilation strategy does not come without risks. Indeed, the resultant decrease in mean airway pressure could cause lung derecruitment, atelectrauma, and biotrauma. Lung collapse and overdistension may also occur simultaneously in severely injured lungs. Interestingly, PEEP was ≤10 cmH_2_O in 77% of the patients in an international survey of ECMO specialists and the ELSO guidelines recommend a modest level of PEEP (10 cmH_2_O) during ECMO support [7]. However, the optimal PEEP in ARDS may vary between patients and depend on several factors (alveolar recruitability, pleural pressure, body weight, and hemodynamics) and may also evolve rapidly during the disease process. Selecting the adequate PEEP for a specific patient and at a specific time point is therefore challenging and a ‘one-size fits all’ strategy would likely not be of any clinical benefit. Several methods have been recently described to guide clinicians in the individualization of PEEP levels during ultra-protective ventilation on ECMO (Fig. 2).

### Electrical Impedance Tomography-Guided Strategy

Electrical impedance tomography (EIT) provides individual, noninvasive, radiation-free imaging of the lungs at the bedside, with global and regional dynamic lung analyses. This technique displays a graphic representation of the regional distribution of lung ventilation and provides real-time information regarding ventilation including heterogeneity of ventilation distribution, regional tidal volume, and gravitational distribution of respiratory system compliance. It identifies impedance changes in the lungs and enables distinction between ventilated and non-ventilated alveolar units. PEEP titration can therefore be guided by EIT, to determine the optimal setting that minimizes lung collapse and overdistension. Franchineau et al. showed the wide diversity in patients’ EIT-derived “best compromise” PEEPs in a series of 15 ECMO patients, with values of 15, 10, and 5 cmH_2_O for 7, 6, and 2 patients, respectively, whereas PEEP 20 and PEEP 0 were never selected [31]. Assessment of the distribution of airway opening and closure by EIT within each lung and between the two lungs is ventilation. Biotrauma AiCLOSE Study (ClinicalTrials.gov Identifier: NCT05196074).

Several limitations of EIT should be mentioned. First, the technique only provides a cross-sectional evaluation of a specific lung region, which may differ from the whole lungs, and only captures the ventral-to-dorsal regional ventilation distribution. Second, it requires specific equipment, which is still not widely available and the acquisition of data is time-consuming. Lastly, there is still a lack of consensus on the EIT target parameters to define the optimal PEEP level. The benefit of such an EIT-guided ventilation strategy to further decrease VILI during ECMO deserves further investigation.

### Transpulmonary Pressure-Guided Strategy

Plateau pressure is a surrogate of the pressure gradient that stresses the lung, i.e., the transpulmonary pressure. As pleural pressure correlates with esophageal pressure, an esophageal manometer can be used to calculate the end-expiratory transpulmonary pressure. This pressure-guided strategy to optimize PEEP can limit atelectrauma and minimize the risk of lung overdistention. It has been used to identify candidates for ECMO (i.e., refractory hypoxemia despite optimal PEEP) [20] or to optimize PEEP on ECMO [32]. In this latter study, patients on VV-ECMO were randomized to either transpulmonary pressure-guided ventilation (n = 52) or a lung rest strategy (n = 52) [32]. The transpulmonary pressure-guided group had a higher rate of successful weaning, a significantly lower 60-day mortality rate (33% vs. 54%, p = 0.03), and shorter ECMO duration (p = 0.004) compared to the lung rest group. However, the transpulmonary pressure-guided strategy remains controversial in patients with ARDS and is not supported by the results of the EPVent-2 trial [33].

### Other Methods

Lung ultrasound can be used to guide the setting of mechanical ventilation in ARDS patients and assess bedside lung recruitment [34]. Changes in the lung ultrasound score correlated with PEEP-induced increases in end-expiratory lung volume in a series of ARDS patients receiving conventional mechanical ventilation [35] and also correlated significantly with computed tomography (CT) scan data in a series of 18 patients receiving ECMO [36].

The recruitment-to-inflation (R/I) ratio is a recent tool that has been developed to evaluate the potential for lung recruitment. It is calculated as the ratio between the compliance of the recruited lung following the application of a high PEEP to that of the respiratory system measured at a lower PEEP. This parameter can be easily measured at the bedside with any ICU ventilator and may help to optimize ventilator settings, particularly PEEP [37]. As of today, this parameter has not been studied during ECMO with very low tidal volume.

## Prone Positioning During ECMO

Prone position is an effective first-line intervention in moderate to severe ARDS [38] that should be considered mandatory before ECMO consideration. However, this procedure during ECMO is still controversial, despite its increasing use, especially during the coronavirus disease 2019 (COVID-19) pandemic [39]. Several observational studies and a recent meta-analysis have shown that prone positioning during ECMO was feasible, safe, and could enhance ECMO weaning and improve outcomes [39, 40]. To date, the lack of RCTs, the fear of accidental decannulation, and the difficulties of routinely training the nursing staff in this procedure are still barriers to generalizing its use in ECMO patients, especially in centers with low ECMO volume. The results of the ongoing randomized controlled PRONECMO trial (ClinicalTrials.gov Identifier: NCT04607551) may help clarify the indications for prone positioning of ECMO patients.

## Gas Exchange Targets on ECMO

There are no evidence-based guidelines for the management of oxygenation, carbon dioxide, or pH in patients with ARDS supported with ECMO, and safe limits of hypoxemia and hypercapnia have not been well established, although both hypoxemia and hyperoxemia have been associated with increased mortality [41]. Gas exchange targets implemented in the EOLIA trial (PaO_2_ 65–90 mmHg; PaCO_2_ < 45 mmHg) [3] are most frequently recommended until more data become available. Because current ECMO membranes allow a significant reduction in mechanical ventilation intensity and can ensure adequate gas exchange despite minimal residual lung function, the ventilator FiO_2_ should be reduced to its minimal value. Additionally, a high fraction of FiO_2_ in lung areas with a low ventilation-perfusion ratio might cause denitrogenation atelectasis, especially if PEEP is low [42]. Lastly, rapid correction of hypercapnia after the initiation of ECMO should be avoided since it was associated with the development of neurological complications [43].

## Mechanical Ventilation During ECMO Weaning

Mechanical ventilation during the weaning of ECMO has received little attention so far. In the EOLIA trial, patients were switched to volume-assist controlled ventilation with tidal volume set at 6 ml/kg when “clinical, radiological, gasometric, and pulmonary compliance had improved” [3]. More recently, in a series of 83 patients undergoing weaning of ECMO, those with higher tidal volume, heart rate, ventilatory ratio, and esophageal pressures swings during a sweep gas-off trial were less likely to achieve safe liberation from VV-ECMO [44]. As mentioned above, prone positioning during ECMO may also facilitate weaning from the device.

## Conclusion

Mechanical ventilation during ECMO for ARDS should aim to reduce VILI by decreasing its intensity. However, further studies are needed to determine how particular ventilator variables should be adjusted during the course of ECMO and during its weaning phase. Pending the results of such studies, EOLIA ventilator settings [3] are a reasonable option.

## Data Availability

Not applicable.
